# Designing Dual-Responsive Drug Delivery Systems: The Role of Phase Change Materials and Metal–Organic Frameworks

**DOI:** 10.3390/ma17133070

**Published:** 2024-06-22

**Authors:** Wanying Wei, Ping Lu

**Affiliations:** Department of Chemistry and Biochemistry, Rowan University, Glassboro, NJ 08028, USA; weiw8@rowan.edu

**Keywords:** stimuli-responsive drug delivery system, phase change material, metal–organic framework

## Abstract

Stimuli-responsive drug delivery systems (DDSs) offer precise control over drug release, enhancing therapeutic efficacy and minimizing side effects. This review focuses on DDSs that leverage the unique capabilities of phase change materials (PCMs) and metal–organic frameworks (MOFs) to achieve controlled drug release in response to pH and temperature changes. Specifically, this review highlights the use of a combination of lauric and stearic acids as PCMs that melt slightly above body temperature, providing a thermally responsive mechanism for drug release. Additionally, this review delves into the properties of zeolitic imidazolate framework-8 (ZIF-8), a stable MOF under physiological conditions that decomposes in acidic environments, thus offering pH-sensitive drug release capabilities. The integration of these materials enables the fabrication of complex structures that encapsulate drugs within ZIF-8 or are enveloped by PCM layers, ensuring that drug release is tightly controlled by either temperature or pH levels, or both. This review provides comprehensive insights into the core design principles, material selections, and potential biomedical applications of dual-stimuli responsive DDSs, highlighting the future directions and challenges in this innovative field.

## 1. Phase Change Materials (PCMs)

Phase change materials (PCMs) are materials that absorb or release a significant amount of “latent” heat when undergoing a change in their physical state, which usually is the change in state between liquid and solid [1]. Throughout the heating or cooling process, PCMs undergo a phase change when the conditions reach a specific temperature [2]. The PCMs’ temperature will remain constant in the process of “latent” heat absorption or release [3]. This phenomenon shows that PCMs can absorb the latent heat and store it, which means PCMs can be chosen as a thermal storage material [4]. PCMs are materials with benefits that include a high capacity for storing solar thermal energy, the capability to transmit large amounts of latent heat, and elevated energy densities [5]. They can be composed of diverse materials, encompassing both natural and synthetic polymers. Encapsulating PCMs enhances the effectiveness of heat transfer and the stability of mechanical systems [6]. PCMs are divided into two types: inorganic phase change materials (IPCMs) and organic phase change materials (OPCMs). Each has its own advantages and disadvantages, and the choice of using them depends on the application’s requirements. IPCMs have been used in thermal energy systems for building applications [7]. IPCMs are further divided into two types: salt hydrates and metallics. Salt hydrates exhibit high energy storage capacity in thermal energy storage (TES) systems [8]. Metallic IPCMs, on the other hand, address certain issues posed by salt hydrates, such as poor thermal conductivity or significant corrosion levels. Metallic IPCMs are better suited for high-temperature applications exceeding 4100 °C [9]. OPCMs typically comprise waxes, polymers, or organic salts, and they are more expensive than IPCMs. Common OPCMs include paraffin waxes and fatty acids such as stearic or palmitic acids [10]. OPCMs find application across various uses, and both paraffin wax and fatty acids demonstrate versatility in terms of operating temperatures. Paraffin wax stands out as a favored OPCM due to its cost-effectiveness compared to many other PCMs. It is characterized by low toxicity, chemical stability, and non-corrosiveness [11]. Therefore, OPCMs like paraffin wax are better suited than salt-hydrate PCMs for TES applications in buildings. TES systems require multiple thermal cycles; however, salt-hydrate PCMs may dissociate during these cycles [12]. Compared to IPCMs, most OPCMs such as paraffin wax, are non-corrosive and chemically stable. They are recyclable and have high latent heat. Unfortunately, they still have disadvantages, such as low thermal conductivity and size increase during the process of phase change [13]. Most of the OPCMs used in TES systems need to be encapsulated in containers. In comparison, IPCMs have the advantages of higher thermal conductivity, low cost, and being nonflammable. Since IPCMs consist mostly of salt hydrates and metallic components, they are corrosive to metals, which means IPCMs have limited long-term applications. Furthermore, IPCMs have a higher risk of phase separation than OPCMs [13,14,15].

### 1.1. Fatty Acid PCMs

**Figure 1:**
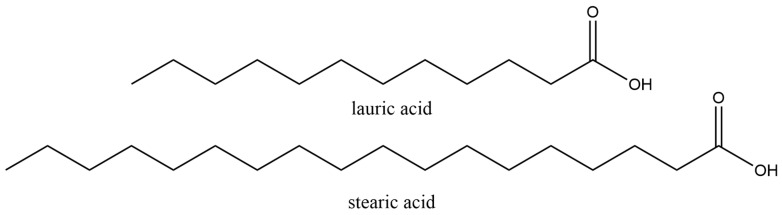
Structure of lauric acid and stearic acid.

Fatty acids, such as myristic acid, capric acid, palmitic acid, and stearic acid, are OPCMs [16,17]. Many researchers have used fatty acids as PCMs and attempted to enhance their thermal properties, including incorporating conducting fillers such as carbon nanotubes (CNTs), metal foams, graphite, etc. For instance, choosing CNTs as a conductive additive in fatty acids helps prevent a reduction in heat storage capacity [17]. Lauric acid is a medium-chain fatty acid (MCFA), defined as a saturated or unsaturated fatty acid with 6 to 12 carbons. MCFAs are typically present in medium-chain triglycerides (MCTs). The melting point of lauric acid is 43.8 °C [18]. These triglycerides are usually hydrolyzed in the gastrointestinal tract, forming free fatty acids through the action of lipases [19,20]. Lauric acid is a saturated fatty acid mostly found in coconut oil. Its chemical formula is C_12_H_24_O_2_, and its chemical structure is shown in Figure 1. Lauric acid (LA) undergoes conversion into monolaurin in the human body. Monolaurin can maintain health by inhibiting pneumococcus and protecting host cells, thereby preventing bacterial infections [19]. Lauric acid, existing as a solid white powder under normal room conditions, finds applications in various industries, including the manufacturing of soaps, cosmetics, and specific food items [21].

**Figure 2:**
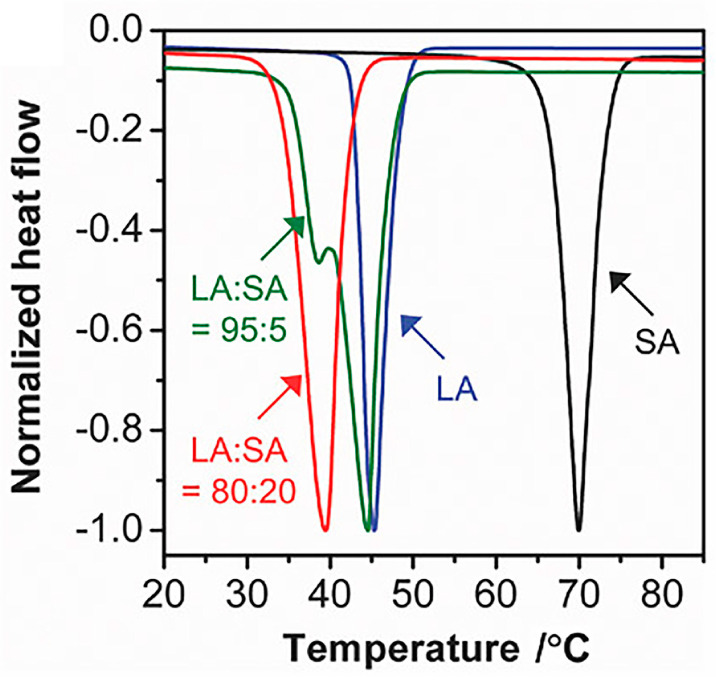
DSC curves of lauric acid, stearic acid, and their mixtures at 95:5 and 80:20 mass ratios [30]. Copyright 2017 Wiley-VCH.

**Figure 3:**
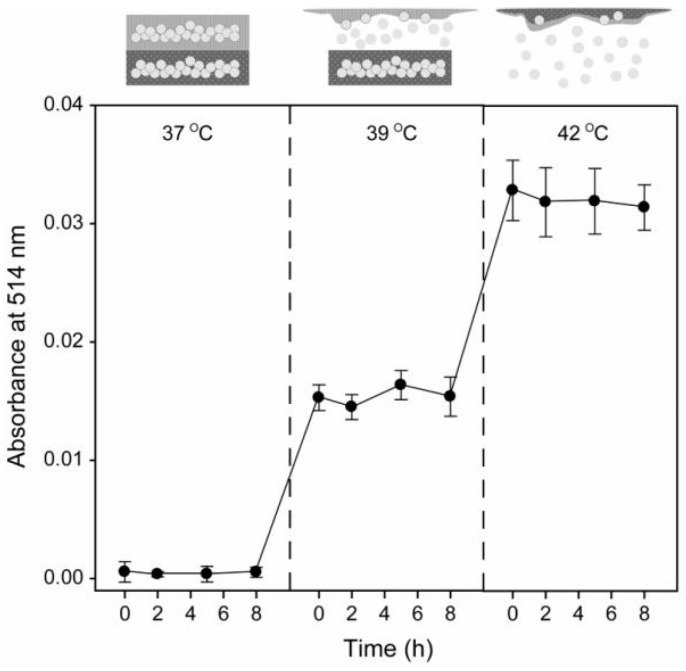
Release profiles of FITC-dextran from gelatin microbeads encapsulated in dual PCMs (i.e., 1-tetradecane and dodecanoic acid) at 37, 39, and 42 °C [32]. Copyright 2010 Wiley.

Stearic acid (SA) is another saturated fatty acid with a total of 18 carbons; the chemical formula is C_18_H_36_O_2_. Stearic acid is synthesized by animal fat hydrolysis or through the hydrogenation of cottonseed or vegetable oil [22,23]. The structure of stearic acid is shown in Figure 2. Both lauric acid and stearic acid are hydrophobic and insoluble in water, but they are all soluble in ethanol [24]. The melting point of stearic acid is around 69.3 °C, and it is non-toxic and biocompatible; it dissolves in common organic solvents. Stearic acid is usually used in the production of soap, detergents, or cosmetics like shampoos [25]. Both lauric acid and stearic acid can be used as phase-change materials [16,26,27,28]. The crystal structure of long-chain fatty acids like stearic acid or lauric acid typically presents a layered arrangement, which can be proven by X-ray diffraction. Its crystalline morphology can be lamellar, acicular, or columnar forms [29]. The mixture of lauric acid and stearic acid (LASA) in an 80:20 ratio gives a melting point of 39 °C. This temperature is slightly above the physiological human body temperature of 37 °C. Figure 3 shows the melting peaks of the mixtures of lauric acid and stearic acid at various ratios in the differential scanning calorimetry (DSC) curve. Based on the finding that LASA in a ratio of 80:20 yields a melting point of 39 °C, it can be used in the drug delivery system as a “gate” for releasing the target drug molecules. The drug molecules encapsulated in the LASA mixture are released as the temperature increases over 39 °C. Several groups have already prepared nanoparticle drug delivery systems with LASA (80:20) for encapsulating doxorubicin (DOX), Rhodamine B (RhB), and IR780 iodide (IR780). The encapsulated drug molecules were not released until the temperature reached above 39 °C [30,31].

### 1.2. PCMs for Drug Delivery Systems

**Figure 4:**
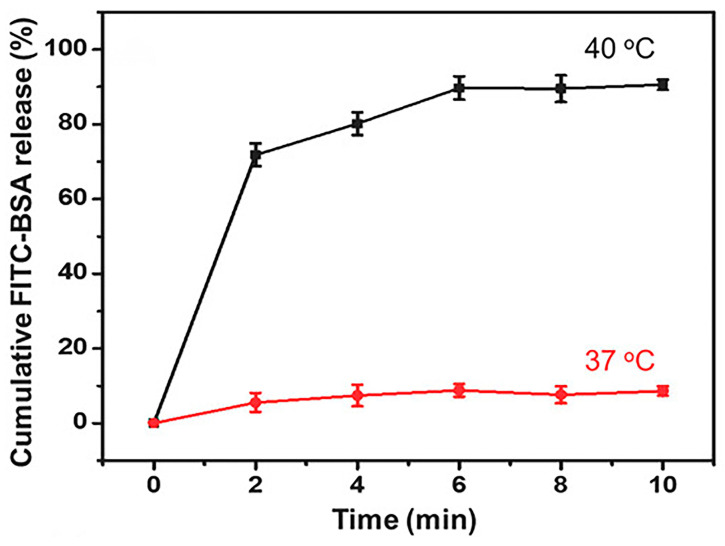
Cumulative release profiles of FITC-BSA encapsulated in LASA microparticles at 37 and 40 °C [36]. Copyright 2018 Wiley.

Stimuli-responsive materials are substances that change their physicochemical properties in response to external factors such as temperature, pH, or light [33,34]. PCMs are stimuli-responsive materials that change their phase states in response to temperature variations. Building upon this, PCMs, such as thermo-sensitive materials, can find applications in drug delivery systems to control release [32]. PCMs are stimulated by temperature to exhibit a phase transition between solid and liquid states. Fatty acid PCMs possess advantages such as temperature sensitivity, low toxicity, and ease of modification. Consequently, they can be utilized in stimulative-drug delivery systems. As the temperature increases above the melting point of the PCMs, they transition from a solid state to a liquid state, and the drug molecules encapsulated in the PCMs are released into the surrounding medium. This simple physical process can be used in the development of a stimulative drug delivery system, allowing for precise control of drug release by adjusting the temperature [28,30,35]. PCMs with phase transitions occurring at slightly above 37 °C are usually chosen for use in temperature-responsive drug delivery systems. For instance, 1-tetradecane, which melts at a temperature range of 38 °C to 39 °C, and lauric acid, with a melting point of 43.8 °C [32], along with LASA in an 80:20 ratio, with a melting point of 39 °C, are noteworthy examples [36]. Figure 4 shows the release profile of dextran encapsulated in a gelatin microbead with a PCM shell (1-tetradecane and dodecanoic acid) at 37, 39, and 42 °C. It demonstrates that there was no release at temperatures below 37 °C. In comparison, the release increased when the temperature reached 39 °C and further doubled at 44 °C compared to that at 39 °C [32].

**Figure 5:**
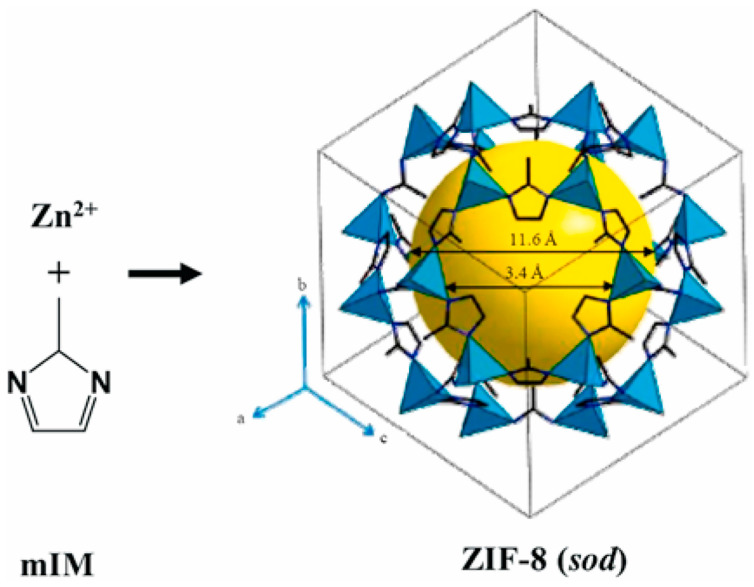
Three-dimensional (3D) structure of ZIF-8 [38]. Copyright 2021 ScienceDirect.

The eutectic mixture of lauric acid and stearic acid in an 80:20 ratio exhibits a melting point of 39 °C. Both lauric acid and stearic acid are naturally biocompatible. LASA can serve as a gating material for precisely controlling drug release [37]. Nanoparticle drug delivery systems can be developed using LASA as a gating material that encapsulates drug molecules in the core. As the temperature increases above 39 °C, LASA undergoes a phase change from solid to liquid, leading to the immediate release of the drug molecules. Microparticles were produced through a coaxial electrospray method, wherein 0.2 g/mL of LASA in ethanol and dichloromethane (DCM) was used as the shell solution, the Rhodamine B (RhB) dissolved in an aqueous gelatin solution as the core solution, and the payload as the bovine serum albumin (FITC-BSA). The cumulative release profiles of FITC-BSA under sustained heating at both 37 °C and 40 °C are shown in Figure 5 [36]. A negligible cumulative release was observed at 37 °C, indicating that the drug molecules were still trapped in the nanoparticles because LASA remained in the solid state. When the temperature reached up to 40 °C, the cumulative release of FITC-BSA reached around 90% within 10 min. This is attributed to the phase change of LASA at its melting point of 39 °C, transitioning from a solid to a liquid state, leading to the release of the majority of FITC-BSA from the microparticles.

## 2. Metal–Organic Frameworks

Metal–organic frameworks (MOFs) are crystalline materials comprising metal ions or clusters coordinated with organic linkers. Most organic linkers typically consist of carboxylic acids or nitrogen-containing ligands [39,40], while the metal constituents or metal clusters often include species such as iron, zinc, cobalt, and copper [41,42,43]. MOFs are unique three-dimensional coordination polymers distinguished by their high porosity [44]. It has different properties from other coordination polymers, the significant voids in MOFs allow for exceptional functionality in applications like gas storage and catalysis. This characteristic enables MOFs to perform significantly in fields ranging from energy storage to drug delivery. Their inherent porosity and structural versatility make them highly promising materials. MOFs exhibit significant internal surface area and tunable pore dimensions. As a result, they find applications in a wide array of disciplines [45,46]. For instance, MOFs constructed with titanium-oxo cluster linkers have been shown to possess 90% porosity [47]. The size of pores is usually below 3 nm, and some large pores reach up to 9.8 nm [46,48]. Also, it has a maximum internal surface area of up to 7000 m^2^/g [45]. Because MOFs contain inorganic and organic components, they have potential applications in clean energy [49], catalysis [50,51] gas separations [38,48], and drug delivery [52]. MOFs are widely used in carbon capture and gas storage; for example, utilizing MOFs to reduce carbon dioxide emissions in coal-fired power plants involves capturing either post-combustion or pre-combustion [53]. MOFs can also be employed for hydrogen storage, for instance, by improving the packing efficiency and volumetric hydrogen storage density through alterations in crystal size distribution and morphological design, thus augmenting the hydrogen storage capacity. Several MOFs have proven to be potential candidates in materials science, transportation engineering, and medical applications. HKUST-1 exhibits a unique structure and performance and is highly suitable for gas adsorption and separation applications [54,55]. MIL-53 has a tunable pore size, making it applicable in catalysis and drug delivery [56,57]. ZIF-8 is pH-dependent, making it valuable for drug delivery [58]. MOF-5, one of the earlier MOFs discovered, finds its primary application in hydrogen storage, holding a pivotal role in the field [59].

### 2.1. Zeolitic Imidazolate Framework-8

**Figure 6:**
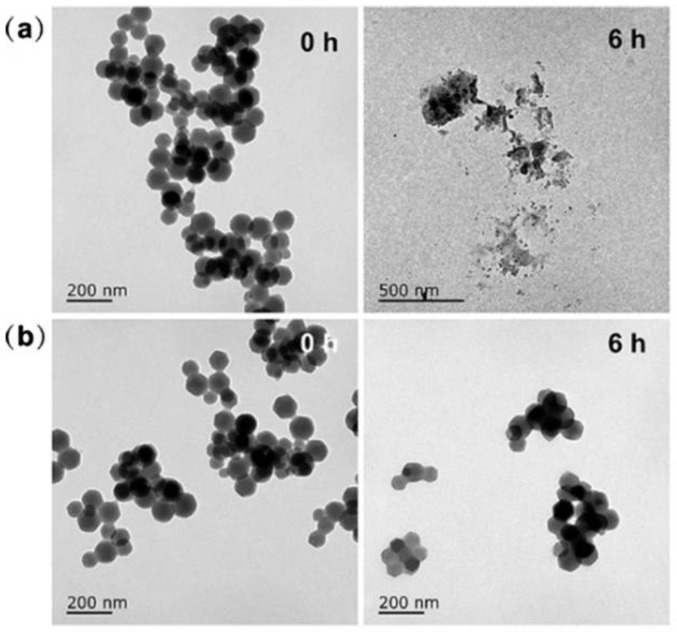
TEM images showing pH-responsive decomposition of DOX/Z-ICG-FA in (**a**) pH = 5.5 PBS and (**b**) pH = 7.4 PBS [74]. Copyright 2022 Springer Nature.

Zeolitic imidazolate framework-8 (ZIF-8) is a classic MOF that can be commercialized with high production. ZIF-8 has many advantages, like a large surface area, controllable porosity, structural tunability, and high thermal and chemical stability. Based on those properties, researchers have modulated ZIF-8 for further exploration and research [60]. ZIF-8 is constructed from zinc ions and 2-methylimidazole (mIM); ZIF-8 is usually described as a three-dimensional network consisting of tetrahedral zinc ions connected with 2-methylimidazole ligands; the structure of ZIF-8 is shown in Figure 6 [61]. The building unit is zinc (II)-imidazolate tetrahedron, a crystal structure with rhombic dodecahedral or cubic shapes [62]. The formation process is similar to synthesizing metal nanocrystals; those nanoparticles are high-symmetry bcc crystal structures [63,64,65,66]. ZIF-8 has the advantage of a high porosity of up to 60% and a pore volume of up to 1.088 mL/g. That property is used for gas storage, absorption, and drug delivery. Researchers prepared ZIF-8 using surfactant-mediated methods by adding Tween 80 or Span 80 to achieve faster adsorption kinetics [67,68]. ZIF-8 has also found application in drug delivery systems due to its high porosity and easy modification. The imidazolate linkers are fundamental organic linkers that render ZIF-8 susceptible to decomposition under acidic conditions. Consequently, ZIF-8 can enable the target drug’s single/multi-stimulus responsive release [69,70]. There are several synthesis methods for ZIF-8, such as the room temperature solution synthesis method [71], solvothermal method [72], and microfluidic synthesis [73]. The most common and convenient method is room-temperature synthesis, achieved by mixing two solutions in a specific ratio and stirring overnight. The solid is then collected, washed, and placed in a vacuum overnight [71].

Researchers have incorporated different base-type additive triethylamines (TEAs) to synthesize ZIF-8 in various sizes [75]. This synthesis method enables the production of ZIF-8 particles with sizes of approximately 134 nm and 288 nm without altering the morphology of the ZIF-8 crystalline structures. After the thermal treatment of ZIF-8, dispersion within a polysulfide (PSf) matrix has been shown to enhance the membranes’ thermal stability and mechanical strength. The modified membrane demonstrated improved performance for CO_2_/CH_4_ separation applications [76]. Carbonization at 800 °C is another effective method to adjust the pore size range for the high-porosity carbon of ZIF-8. This method increased the exposed surfaces with nitrogen-containing functional groups, which increased the CO_2_ adsorption capacity [68]. Those methods make it easier to add additives. The chemicals commonly used in the synthesis are zinc nitrate hexahydrate and 2-methylimidazole. The reagents, molar ratio, solvent use, and condition are different depending on the needed properties of ZIF-8 [77]. The synthesis temperature plays a vital role in determining the size of ZIF-8. For instance, choosing 1-methylimidazole and zinc acetate hexahydrate in a methanol system produced rhombic dodecahedra ZIF-8 (300 μm) under solvothermal conditions. Conversely, using the same chemicals but different synthesis conditions at room temperature, ZIF-8 assumes a truncated rhombic dodecahedra shape with an average size of 3 μm [78]. Furthermore, variations in chemical composition induce structural changes in ZIF-8. A truncated cube shape in ZIF-8 (180 nm) was obtained using zinc acetate hexahydrate and 2-methylimidazole coupled with cetyltrimethylammonium bromide (CTAB) as a surface-specific capping ligand via a hydrothermal synthesis in water [65,79]. In contrast, a rhombic dodecahedra-shaped ZIF-8 (660 nm) was synthesized using zinc nitrate dihydrate and 2-methylimidazole without CTAB [80].

### 2.2. ZIF-8 for Drug Delivery System

All ZIFs contain transition metals and imidazole ligands; ZIF-8 comprises zinc ions with tetrahedral coordination linked by imidazole ligands. ZIF-8 has many advantages for applications in drug delivery systems. The imidazole ligands can deprotonate and generate an anionic multi-terminal ligand, exhibiting strong alkalinity. When these imidazole ligands interact with metal ions, they form a coordination of a certain intensity. The choice of transition metal ions, such as Mn^2+^, Co^2+^, Cu^2+^, and Zn^2+^, is based on their suitable softness and hardness. The reversible coordination between transition divalent metal ions and organic ligands provides the constructed MOFs with a distinctive advantage in drug delivery [52,69,81]. Another reason for selecting MOFs as drug carriers is the consideration of toxicity and its intensity. Both the metal ions and the organic ligands must exhibit good biocompatibility, and it is imperative to avoid highly toxic metal ions such as Cr and Ni. The most suitable metal ions, such as Fe, Zn, and Mn, should be essential elements for human life activities [69].

**Figure 7:**
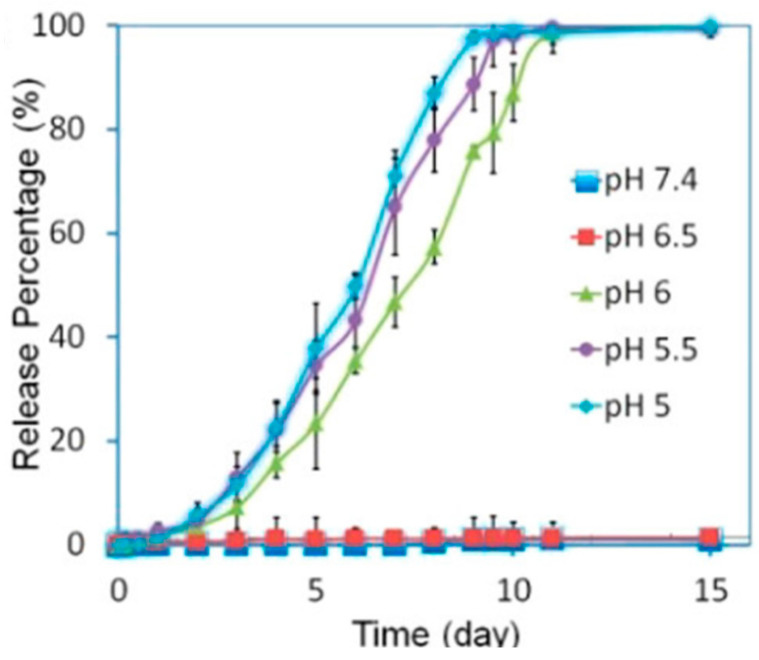
The pH-responsive release profile of DOX from DOX@ZIF-8 particles [88]. Copyright 2016 American Chemical Society.

The imidazole linkers have strong alkalinity, ZIF-8 decomposes during acid conditions, and ZIF-8 has been proven as a pH-responsive material, as Figure 7 shows below; it shows Dox-loaded-ZIF-8 with an ICG solution immersed in pH = 5.5 PBS and pH 7.4 PBS for various periods of time. The ZIF-8 decomposed after six hours in pH 5.5 compared with the pH 7.4 buffer [74]. The drug delivery system, with the capability to encapsulate and release drugs in response to an acidic environment, has become one of the most extensively researched domains [82]. The blood and normal tissue pH is typically around pH 7.4. In contrast, the pH in tumor tissue tends to be more acidic, ranging from pH 5.5 to 6.0 [83]. The reason for using the pH-responsive drug carrier is to reduce the premature release of the drug during transportation in the blood circulation. This improvement can enhance the effective release of anticancer drugs in the tumor tissue or the target organ [84,85]. Excessive drug release during transportation can cause undesirable side effects, and insufficient drug release within target organs or cells hinders the achievement of efficient therapy [86].

**Figure 8:**
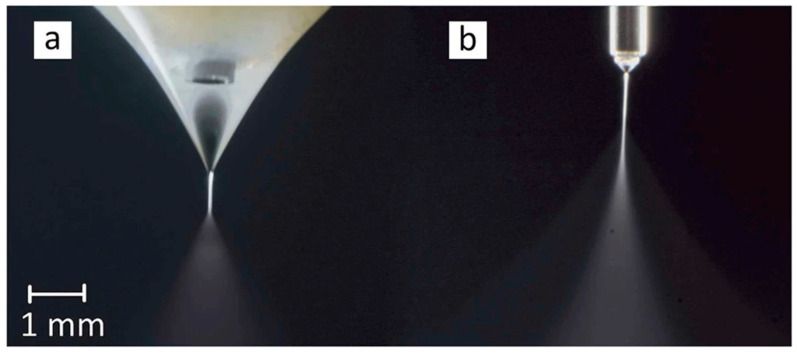
The Taylor cone of ethanol electrospraying at (**a**) flow rate = 5 mL/h and V = 7.3 kV and (**b**) flow rate = 2 mL/h and V = 4.3 kV [89]. Copyright 2016 Nature.

Two methods are currently used to encapsulate drugs in ZIF-8. The first method is post-synthetic encapsulation, which involves synthesizing ZIF-8, preparing the stock solution with drug molecules, adding ZIF-8 to stir for days, removing the suspension, collecting the precipitation in a centrifuge machine, and drying it in a vacuum [87]. The second method is the one-pot synthesis method, which involves preparing zinc acetate hexahydrate and a 2-methylimidazole solution separately. Firstly, the drug stock solution is added to the zinc acetate hexahydrate solution and mixed well. Subsequently, the 2-methylimidazole solution is added dropwise to the mixture. Lastly, all the precipitate is collected using a centrifuge machine and dried in a vacuum [88]. Figure 8 shows the release profiles of ZIF-8 encapsulated with doxorubicin using the one-pot synthesis method under different pH conditions. The release percentage reached 100% within the pH range of 5–6, compared to 0% release within the pH range of 6.5–7.4. This result substantiated the pH-responsive nature of the drug-loaded ZIF-8 carrier, demonstrating that the one-pot synthesis method effectively encapsulated drug molecules into ZIF-8 [88].

## 3. Coaxial Electrospray

Electrospray (ES) is widely used in analytical chemistry and material science to prepare nanoparticles. During electrospray, liquid samples form charged droplets under high voltages. The electrospray is commonly used to produce nanoparticles, typically in the range of 1 to 100 nanometers [90]. ES, also called electro-hydrodynamic atomization, has become popular in the fabrication of nanoparticles and thin films. With the discovery of electrospray ionization in the 1980s, more people became interested in the method. In 2002, John Bennett Fenn won the Chemistry Nobel Prize for discovering that ES ionization could be used in mass spectrometry [91,92]. Electrospinning and electrospraying are similar technologies; depending on the liquid used in the experiment, electrical energy can yield fiber (electrospinning) or particles (electrospraying). The electrospraying and electrospinning experiment setups are the same, including a high-voltage supply, a syringe pumper, a syringe filled with solution, a metal needle, and a conductive collector [93].

The coaxial ES (Co-ES) can produce micro- or nano-sized particles in multilayers by introducing coaxial solution jets. Compared to alternative microencapsulation methods, the benefits of choosing this technique include superior encapsulation efficiency, bioactivity, and uniform size distribution [94]. The encapsulation efficiency of particles reached up to 100% in a core–shell spherical shape. The size range of multilayer particles ranges from 10 nm to 100 μm depending on the electric field between the needle tips and the ground. The “Taylor cone” is formed in an inverted triangle shape due to the repulsive force caused by the electric field, which elongates the core and shell liquids. The jet of solution at the end of the Taylor cone splits into multilayered droplets due to the repulsive force [95,96,97]. The uniaxial electrospray uses only one solution, while the Co-ES is modified through uni-axial electrospray by attaching a coaxial needle to introduce two solutions separately. The common solvents used in electrospray include water, ethanol, dichloromethane, and dimethylformamide, which are volatile solvents. Core–shell nanoparticles with a hard shell can be produced with polymers like gelatin [98], Poly(lactic-co-glycolic acid) [99], and Polylactic Acid [100] as the shell solution [95].

**Figure 9:**
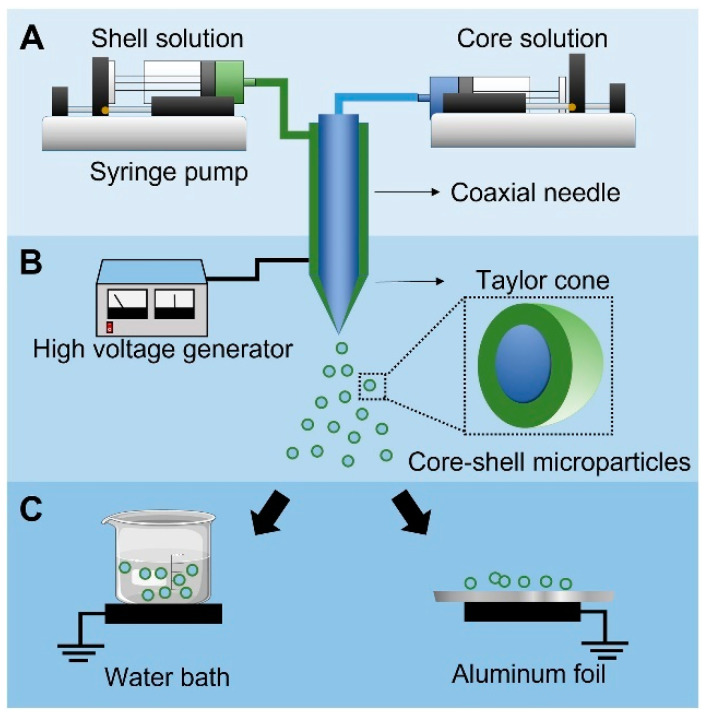
Schematic illustrating Co-ES setup. Step (**A**): Polymer solutions for the shell and drugs for the core are loaded into the respective outer and inner sections of a coaxial needle, which is connected to syringe pumps that control the jetting speeds of the solutions. Step (**B**): Upon application of high voltage, the charged solutions form a stable double-layered conical structure (i.e., Taylor cone) at the needle tip, subsequently breaking into core-shell droplets. Rapid solvent evaporation in air results in the formation of dense microparticles with a core-shell structure. Step (**C**): The microparticles are collected using either aluminum foil or a water bath [105]. Copyright 2022 ScienceDirect.

Many parameters and factors affect the final products of Co-ES. These include the flow rates of the inner and outer solutions, viscosities, surface tensions, relative humidity, surrounding temperature, and electric intensity. The applied voltage is the main factor that affects the morphology of particles. The higher the voltage applied to the needles and collector, the greater the electric intensity between tips and collectors. This causes the jets to split into multi-jets, forming finer particles. Figure 9 shows two voltages from left to right with the increased applied positive voltage of 7.3 kV and 4.3 kV [89]. The formation of the Taylor cone shape is shown during the process of increasing voltage. When the voltage reached a significantly high level, a phenomenon known as “multi-jet” appeared. This indicates the generation of multiple liquid jets that split, resulting in smaller and more dispersed particles. As a result, the speed and distribution of particles increase [101,102,103]. In addition to the applied voltage, the flow rates of the core and shell solutions can also influence the Co-ES products. As the flow rate decreases, the droplet size also decreases. This phenomenon occurs because less electric force is needed to overcome the hydrodynamic forces at lower flow rates [104].

The materials used in Co-ES play a vital role in influencing surface tension, viscosity, and electrical conductivity. Electrical conductivity is related to the liquid jet’s stability; adjusting the core liquid’s electrical conductivity helped achieve a stable Taylor cone [106,107]. The purpose of using Co-ES is to form core–shell nano-/microparticles. To form particles, the outer solution needs to be completely coated on the inner liquid, and a stable liquid jet can be achieved by adjusting the surface tension by adding surfactants such as Tween 80 or polyvinyl alcohol (PVA) [97,108,109]. Viscosity is the measure of a fluid’s resistance to flow, reflecting the strength of internal molecular interactions. In Co-ES, the liquid can transfer electrical stress through viscosity to form a stable cone-shaped jet. The shell liquid should have sufficient viscosity to coat the core. The low viscosity in the shell liquid makes forming the cone jet difficult. However, suppose the viscosity of the shell liquid is too high. In that case, a significantly large electric field is required to overcome the high viscosity and drag the liquid out for cone-jet formation [102,107,110]

### 3.1. Co-ES Setup

**Figure 10:**
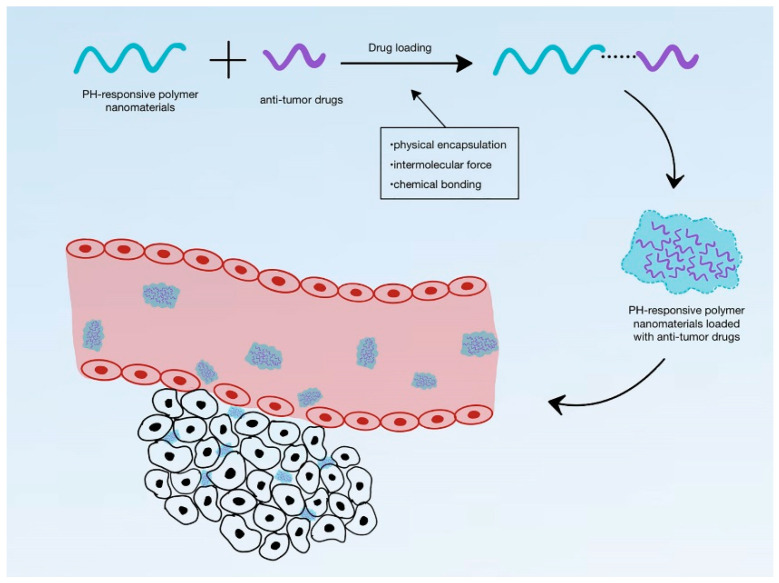
Schematic diagram showing loading of anti-tumor drugs into pH-responsive materials and their delivery to tumor tissues [122]. Copyright 2022 Frontiers Media S.A.

Co-ES needs two syringes loaded with two solutions. The voltage power supply connects the positive to the spinneret and the ground to the collector. The syringe fills with a solution that can produce particles, usually with a polymer solution and additives. The solution is squirted at a constant flow rate with the syringe pump; the syringe pump can adjust the flow rate. In near-field electrospraying/electrospinning, 5–25 kv is usually applied to the spinneret, and the distance between needles and the collector is 5–25 cm [93,111,112]. The polymers should have sufficient viscosity to produce the particles via ES [113,114]. The Co-ES can produce micro- or nanoparticles with multiple layers with more than one solution. The Co-ES has the potential advantage of increasing the encapsulation effectiveness, injecting diverse additives into particles, and adjusting the morphology of particles. In a typical Co-ES process, the polymer solution is loaded into the syringe and attached to the pump. The spherical polymer solution exits through needle tips when the voltage is not applied. After the voltage is applied to the metal tips, the spherical shape turns into the cone shape of the droplets due to the repulsive force between the positive charges at the polymer droplet’s surface and the conductive collector; the cone shape is called Taylor Cone [94,115]. Figure 10 shows that a low-concentration polymer liquid in a high flow rate forms very tiny liquid droplets under a high voltage (15–25 kV). The products on the collector are micro- or nano-sized particles of various sizes and shapes. The morphology of products can be controlled through the ES parameters to produce solid or porous particles [116]. The electrical field elongates the core and shell solution at the tips of the metal needle to become a cone shape [117]. This phenomenon helps control the morphology of polymer particles in organic solvents. The controlled morphology of particles at micro- or nanoscales can be used in many applications. These particles can encapsulate various substances, including food additives [118], drugs [119], and functional materials [120]. The Co-ES can adjust the particle size range to achieve optimized encapsulation [95]. Also, in order to control the diameter of the spray, a metal ring connected to a voltage supply was placed in the path of the ES jet [121].

### 3.2. Nano-/Microparticles for Drug Delivery Systems

Active pharmaceutical ingredients (APIs) are used in medicine to treat diseases, making the precise delivery of APIs to the human body important [123]. Drug delivery systems (DDSs) have various delivery methods, including oral, nasal, injection, and inhalation [124]. Challenges for DDSs include controlling drug loading, reducing side effects, addressing random distribution, and preventing drug accumulation in the body [125]. Controlling the concentration of therapeutic compounds is crucial for minimizing side effects and toxicity. In recent years, the controlled-release DDS has gained tremendous attention for its ability to improve the effectiveness of drug delivery by enhancing control over the release process [126,127]. This goal can be achieved by designing carriers that deliver precise amounts of the target drug. Nano-/microparticles are widely used in DDSs, enabling the controlled release of therapeutic agents to specific target organs. They offer benefits such as reduced side effects, controlled dosage, and precise release percentages. Many nano-/microparticles have been chosen as drug delivery carriers like polymers [128], silicon or carbon material [129,130], liposomes [131], and magnetic materials [132,133,134]. Currently, nanotechnology has rapidly advanced in the treatment of diseases such as lung cancer [135], breast cancer [136], atherosclerotic cardiovascular disease [137], and brain cancer [138]. The advantages of using nano-/microparticles as drug carriers include their biocompatibility, efficient drug loading, and biodegradability [139,140]. Data have proven that the combination of proteins with nanomedicines facilitated the assembly of protein subunits to deliver drugs on-site to specific tumors [141,142]. Nanoliposomes, composed of bilayer lipids containing an aqueous reservoir, are used in DDSs. It has been chosen to deliver hydrophilic and hydrophobic drugs since many anticancer drugs are hydrophobic compounds. Nanoliposomes serve as suitable carriers for their dissolution without the use of harmful organic solvents [143,144].

As nano-/microparticles are developed as biocompatible carriers for DDSs, the synthesis method for producing nano-/microcarriers with drugs becomes crucial. Co-ES can generate spherical-shaped particles with various cores/shells, enabling the encapsulation of multiple drugs in the core [95,145]. Extensive studies have explored this aspect by controlling the constant flow rate of the core and shell solution to produce particles with different drugs. The objective is to regulate the release rate of drugs [146]. The common materials used to create a multilayer hard shell include poly(lactide-coglycolide) (PLGA) [147], poly(lactide) (PLA) [148], and gelatin [98]. The advantage of Co-ES lies in its ability to preserve the bioactivity of the payload, making it suitable for encapsulating proteins, antibodies, and sensitive drugs [96,149]. Furthermore, Co-ES can effectively encapsulate drugs, achieving a high encapsulation rate of up to 75% for estradiol-loaded PLGA capsules [145]. By adjusting the parameters and surrounding environment of Co-ES, precise control over the drug release rate has been achieved, thereby meeting specific therapeutic requirements [94,150].

## 4. Stimuli-Responsive Drug Delivery Systems

Drug delivery systems (DDSs) can be categorized into various types based on their characteristics and applications, including targeted DDSs, transdermal DDSs, oral DDSs, responsive DDSs, etc. [151,152,153,154]. Stimuli-responsive DDSs have unique characteristics, which can deliver loaded drugs with control of dosing and release time, responding to both exogenous and endogenous stimuli [155]. A stimuli-responsive DDS’s advantage is its capability for drug release under various external or internal stimuli, even achieving dual/multiple responsiveness. This DDS can efficiently control the loading of the dose and sustainably release the drug. So far, many types of stimuli-responsive DDSs have been developed and researched, including temperature, light, pH, magnetic, and electrical responses [156,157,158,159,160].

**Figure 11:**
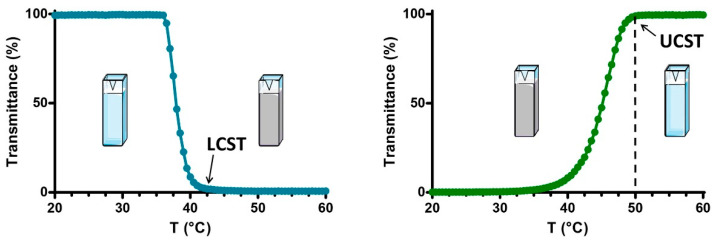
Transmission curves of the temperature-responsive polymer at different temperatures, the LCST (lower critical solution temperature), and the UCST (upper critical solution temperature) [165]. Copyright 2019 ScienceDirect.

Endogenous stimuli include both internal and biological stimuli. In this type of DDS, the synthesis of nanocarriers requires an appropriate material that responds to specific endogenous stimuli, causing the structure to break and facilitating immediate drug release. For instance, pH-responsive DDSs release drugs when the pH reaches a specific value, breaking the structure to release the drug into the surroundings (Figure 11). pH-responsive DDSs are capable of facilitating therapeutic release inside cells and tissues. For example, tumor tissue has a slight pH difference compared to normal tissue, allowing nanocarriers to release drugs in the tumor tissue while maintaining the intact structure in normal tissues. Common pH-responsive materials include liposomes, polymers, carbons, etc. Other endogenous stimuli include enzyme responsiveness and redox responsiveness [122,155,161,162,163,164].

**Figure 12:**
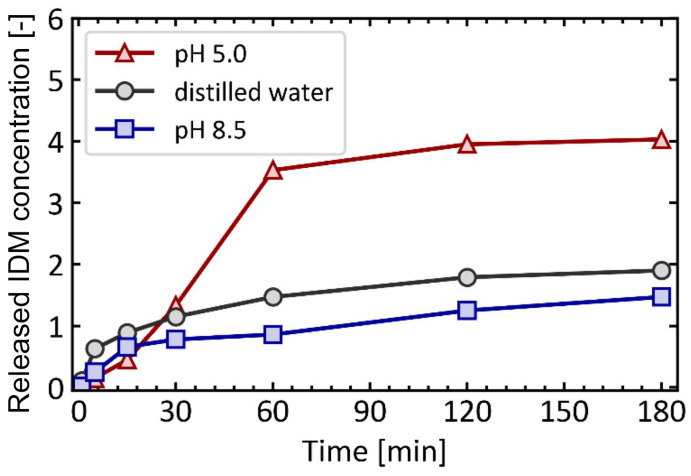
Release profiles of IDM from ZIF-8 at different pH conditions [173]. Copyright 2022 ScienceDirect.

Exogenous stimuli include temperature, magnetic fields, UV light, and electric fields. These disrupt the structure of the selected nanocarrier and release the loaded drug into the targeted tissue [166,167,168]. For example, temperature-responsive DDSs can keep the drug at normal physiological temperature within specific nanocarriers but release the drug when the temperature reaches a threshold value in diseased tissue. Some diseased or tumor tissues often have higher temperatures, approximately around 40 °C to 42 °C or even higher [169,170]. Poly(N-isopropyl acrylamide) (PNIPAM) is a potential material for thermo-responsive DDS. It forms a hydrophobic globule when the temperature exceeds the low critical solution temperature, causing drug release due to hydrophobic interactions. However, if the temperature drops below 32 °C, the PNIPAM coil exhibits different solubility in water [171,172]. Figure 12 illustrates the transmittance curves of PNIPAM under varying temperatures. On the left, the curve represents the status of polymers near their lower critical solution temperature (LCST). As the temperature increases, the transmission decreases, indicating a transition from a liquid-like to a solid-like state. Conversely, on the right, the curves show the behavior of polymers near their upper critical solution temperature (UCST). As the temperature rises, the transmittance increases, suggesting a transition from a solid to a liquid-like state [165].

### 4.1. pH-Responsive DDS

**Figure 13:**
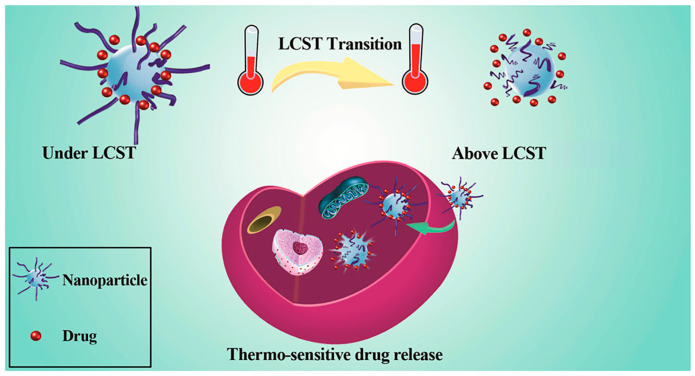
Drug release under/above LCST in a temperature-responsive manner [184]. Copyright 2016 American Chemical Society.

A pH-responsive drug delivery system is an endogenous stimuli-responsive DDS. The drug release depends on the pH of organs, tissues, or cells. pH values are suitable stimuli for controlled drug release due to the variations in pH values among different tissues, organs, and cells [174]. This property enables the controlled delivery of drugs at specific sites and times, thereby enhancing the effectiveness of therapy and improving accuracy. For example, the pH value is different in the tumor (6.4–7.0), stomach (1.5–3.5), small intestine (5.5–6.8), colon (6.4–7.0), and lysosome (4.5–5.0) [175,176]. Inflammatory tissues and tumors have lower pH values than blood and normal tissues. The physiological pH values for humans are typically around pH 7.4 [177,178]. Hydrogels have been proven to be pH-stimuli-responsive materials, playing a crucial role in biomedical fields. They are water-based materials with high water percentages and water retention. Additionally, hydrogels have similar physical properties to living tissues. They exhibit responsiveness to changes in the acidity or alkalinity of the surrounding environment [174,179]. A dual-stimuli-responsive DDS can be obtained using hydrogels, such as through the addition of a second temperature-responsive material or a redox stimuli-responsive material. For example, the copolymer poly (N-isopropyl acrylamide-co-propyl acrylic acid-co-butyl acrylate) was synthesized as a hydrogel substrate. This material exhibited a liquid state at pH 7.4 and 37 °C, transitioning into a physical gel state under conditions of pH 6.8 and 37 °C [180]. Inorganic pH-responsive materials have more significant advantages in biocompatibility, modification, and thermal stability compared to organic materials. The pH-responsive inorganic materials include carbon-based nanostructures, mesoporous silica, and calcium silicate-based materials [162,181,182]. Calcium phosphate-based nanomaterials are suitable as pH-responsive materials due to their high biocompatibility. Under acidic conditions, calcium phosphate-based materials decompose into Ca^2+^ and PO_4_^3−^ ions, the body’s fundamental elements [174,183]. Figure 13 presents the release profile of indomethacin (IDM) encapsulated in ZIF-8 under varying pH conditions. IDM was used as a model drug, and its release was measured at each time point and recorded in the release profile. The data clearly show that the highest release occurs in the acidic environment at pH 5.0, while the release in the alkaline environment is lower than that in the neutral environment [173].

### 4.2. Temperature-Responsive DDSs

Temperature-responsive DDSs, one of the exogenous-stimuli-responsive DDSs, use nanocarriers to control the release of drug molecules at varying temperatures. Temperature is applied to the nanoparticles as an external stimulus. When a pathological lesion naturally increases the temperature or external factors elevate the temperature, the drug is released to exert its therapeutic effect. The LCST refers to a specific temperature at which a polymer material dissolves through phase separation and transitions into a solution [185]. This property is unique to polymeric materials. Many temperature-responsive nanoparticles have been designed based on this characteristic [184]. Temperature-responsive nanoparticles have been constructed from metals, carbons, liposomes, carbon nanotubes (CNTs), and magnetic materials [186,187]. To date, many biocompatible temperature-responsive polymers have been used for DDSs. The core requirement is that the temperature-responsive material is able to release the drug with a slight temperature change [172,188]. Several promising materials include poly(N-isopropyl acrylamide) (PNIPAM) derivatives [189], layer-by-layer (LBL)-assembled nanocapsules, and lauric acid–steric acid mixtures [30,188].

**Figure 14:**
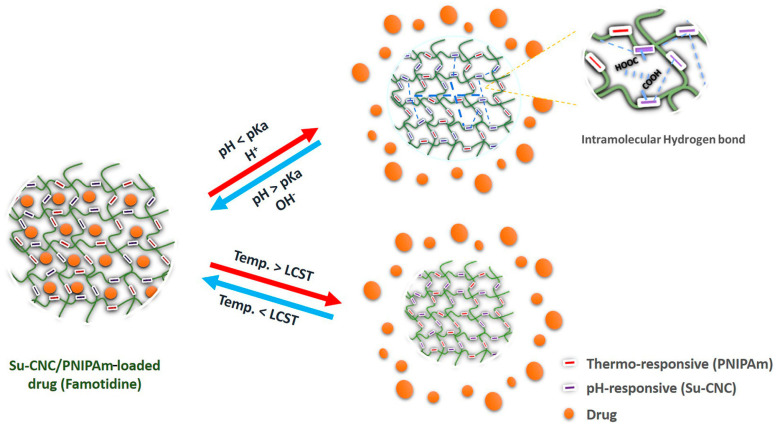
Schematic diagram of Su-CNC/PNIPAM drug release behavior in response to varied temperature and pH conditions [197]. Copyright 2022 ScienceDirect.

Common methods include phase separation, co-precipitation, and electrospray to synthesize temperature-responsive nanoparticles [190,191,192]. The temperature-sensitive DDS finds applications in the delivery of anticancer medications and imaging agents [193,194]. The advantages of temperature-responsive DDSs include low toxicity, prevention of overdosage, and more controllable drug release [195]. Hydrogels have been extensively used in biomedical applications, such as wound healing. These materials show temperature-responsive characteristics, undergoing a solid-to-gel transition in response to temperature changes [196]. PNIPAM is used as hydrogels with significant potential for biomedical applications. Formulating PNIPAM with other functional components can maximize its effectiveness and avoid disadvantages, such as low drug loading capacity. PNIPAM exhibits hydrophilicity below its LCST and transitions to hydrophobicity above the LCST, as shown in Figure 14. Additional stimulus materials can be introduced to achieve a multi-stimuli DDS [184].

### 4.3. pH/Temperature Dual-Responsive DDSs

**Figure 15:**
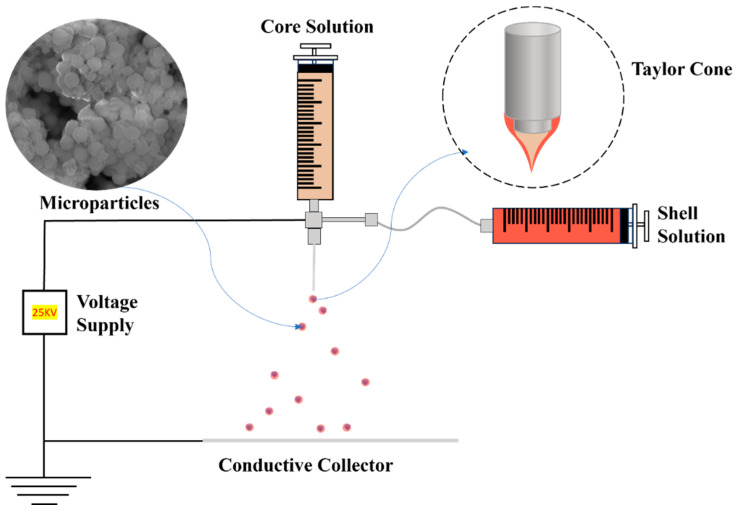
Schematic illustration of Co-Es to produce core–shell microparticles.

Based on pH- and temperature-responsive materials, dual or multiple responsiveness can be established. The pH/temperature dual-responsive DDS is designed to respond to changes in the pH and temperature of the surrounding environment, providing a novel approach to controlling drug release. The dual-responsive DDS can be designed to create a more precise drug control system, improving targeted and gradual drug release. This system also holds the potential to reduce side effects and increase the therapeutic efficacy of drugs. Hydrogels, as pH-responsive materials, undergo a solid–gel transition with changes in pH. Hydrogels can absorb and retain a large volume of aqueous content as biodegradable materials. They can be used to design a pH/temperature dual-responsive DDS. The temperature-responsive PNIPAM is suitable for combining with hydrogels. Succinylated cellulose nanocrystals (Su-CNC) are synthesized by incorporating hydrogels and PNIPAM through free radical polymerization reactions. At temperatures of 35 °C and above, Su-CNC showed responses to temperature changes, resulting in swelling and increased hydrophobicity, consequently leading to hydrogel shrinkage. Moreover, a notable change in PNIPAM occurred when the pH shifted from 8 to 2 [197]. The LCST of PNIPAM is around 32 °C, which is close to physiological body temperature, making it an ideal material for DDSs. When the temperature decreases to the LCST, the drug remains encapsulated within the crystal, as illustrated in Figure 15. However, upon temperature increase beyond the LCST, PNIPAM transitions to a hydrophilic state. Additionally, at elevated pH levels, the polymers swell, causing the drug to be retained within crystals. Figure 15 demonstrates that drug release occurs only when the temperature increases and the pH decreases [197,198,199].

## 5. Summary and Outlook

This review has explored the intricate design and application of stimuli-responsive DDSs that leverage the unique properties of PCMs and MOFs facilitated by coaxial electrospraying. We discussed the fundamental aspects of PCMs, focusing on their ability to undergo physical state changes at predefined temperatures, which is critical for controlled drug release. Specifically, the use of lauric acid and stearic acid as PCMs in DDSs exemplifies how these materials can act as thermal gates, melting slightly above body temperature to release encapsulated drugs. In parallel, the properties and functionalities of MOFs, particularly ZIF-8, were detailed, highlighting their stability under physiological conditions and responsiveness to acidic environments, which are beneficial for targeted drug delivery. The integration of these materials through coaxial electrospraying allows for the fabrication of core–shell structures that encapsulate drugs within MOFs surrounded by PCM layers, ensuring controlled release in response to temperature and pH stimuli.

Looking forward, the development of dual-stimuli DDSs presents several avenues for further research and optimization. The potential to refine the coaxial electrospraying process to enhance encapsulation efficiency and payload capacity is vast. Future studies could explore the incorporation of multiple responsive materials to address a broader range of physiological signals, such as redox potential and enzymatic activity, which could lead to more precise targeting and release mechanisms. Additionally, the environmental stability and reusability of these systems need a thorough investigation to assess their long-term performance and viability in clinical settings. As the field progresses, regulatory considerations and scalability of production will become increasingly important in transitioning from laboratory research to practical applications. Collaborative efforts between chemists, material scientists, and biomedical engineers will be pivotal in overcoming these challenges and realizing the full potential of advanced drug delivery systems.
